# Point-of-care ultrasound of the diaphragm in a liver transplant patient with acute respiratory failure

**DOI:** 10.1186/s13089-015-0021-9

**Published:** 2015-03-28

**Authors:** Federico Barbariol, Luigi Vetrugno, Livia Pompei, Adelisa De Flaviis, Giorgio Della Rocca

**Affiliations:** Department of Anesthesia and Intensive Care Medicine, University of Udine, P.le S. M. della Misericordia 15, 33100 Udine, Italy

**Keywords:** Ultrasonography, Point-of-care, Diaphragm dysfunction, Acute respiratory failure, Liver transplantation, Intensive care, Anesthesia

## Abstract

In some intensive care, nowadays, ultrasound diagnostics have become an extension of the physical examination (like a stethoscope). In this report, we discuss the case of an acute respiratory failure which arose immediately after the end of general anesthesia. An initial bedside ultrasound evaluation applying the ‘BLUE protocol’ showed no pathological changes capable of explaining the clinical picture; however, by evaluating also the right and left hemidiaphragms, we made a diagnosis of diaphragmatic dysfunction, which would probably have been difficult to diagnose without the aid of the diaphragm ultrasound. We therefore decided to avoid intubation, transfer the patient to the intensive care unit, and treat him conservatively with non-invasive ventilation only. To our knowledge, this is the first case report that has shown the usefulness of ultrasonography in detecting diaphragmatic dysfunction as a cause of acute respiratory failure with a subsequent change in patient management. The use of bedside ultrasonography provides practical functional information on the diaphragmatic function in patients with acute respiratory failure and can also be easily repeated if follow-up is required. This feature is still held in little consideration, but it can affect the diagnosis and the treatment of critically ill patients.

**Electronic supplementary material:**

The online version of this article (doi:10.1186/s13089-015-0021-9) contains supplementary material, which is available to authorized users.

## Background

In some intensive care, nowadays, ultrasound diagnostics have become an extension of the physical examination (like a stethoscope) [1]. In the clinical practice of our intensive care unit (ICU), we perform a whole echocardiography every morning (echo-round) and every time a patient is admitted. Clinical studies have shown that diagnostic ultrasound can be superior to the physical exam alone [2,3]. One of the most successful approaches in the field of acute respiratory failure is the ‘BLUE Protocol’ [4], but it does not address the issue of diaphragm dysfunction (DD). It may be due to several causes such as trauma, surgery, myopathy, neuropathy, mediastinal masses, mechanical ventilation, and diseases that cause lung hyperinflation and also to metabolic, infective, or inflammatory disorders. These alterations may affect only one or both hemi-diaphragms and may present with different intensities, ranging from only a partial loss of the ability to generate negative pressures (weakness) to a complete loss of diaphragmatic function (paralysis) [5]. In the setting of liver transplantation (OLTx), right diaphragmatic dysfunction is an occurrence often overlooked but actually quite common in the immediate postoperative period: McAllister [6] found that 79% of liver recipients had right phrenic nerve injury and approximately half of these patients also had hemidiaphragm paralysis. Patients with unilateral diaphragmatic paralysis are usually asymptomatic, and phrenic nerve conduction generally tends to recover within a few months [7]. Most of these patients are able to maintain adequate ventilation and gas exchange both at rest and during mild exercise, probably through compensatory mechanisms such as an increase in the work of the normal hemidiaphragm and of the intercostal muscles [8]. The combination of anesthesia and surgical insult can induce changes in respiratory mechanics on their own, such as hypoxemia, reduced lung volume, and atelectasis, then leading to a restrictive syndrome. If in this context you put a diaphragmatic dysfunction too, respiratory function may be so impaired as to lead to respiratory failure after discontinuation of mechanical ventilation [9].

## Case presentation

**Figure 1:**
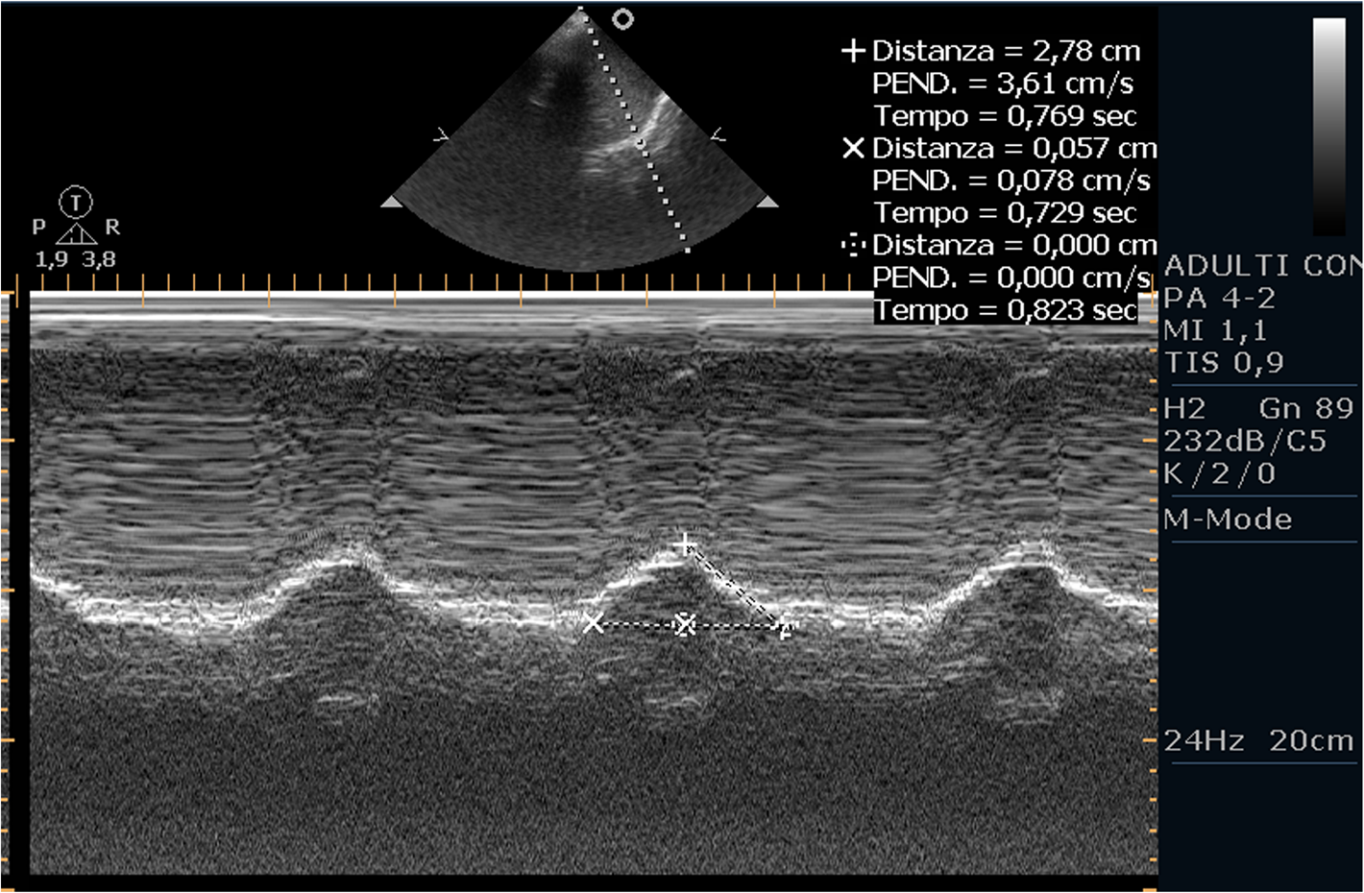
**The normal left hemidiaphragm of the patient.** We can see the diaphragmatic excursion showing an inspiratory peak of 2.78 cm above the baseline.

**Figure 2:**
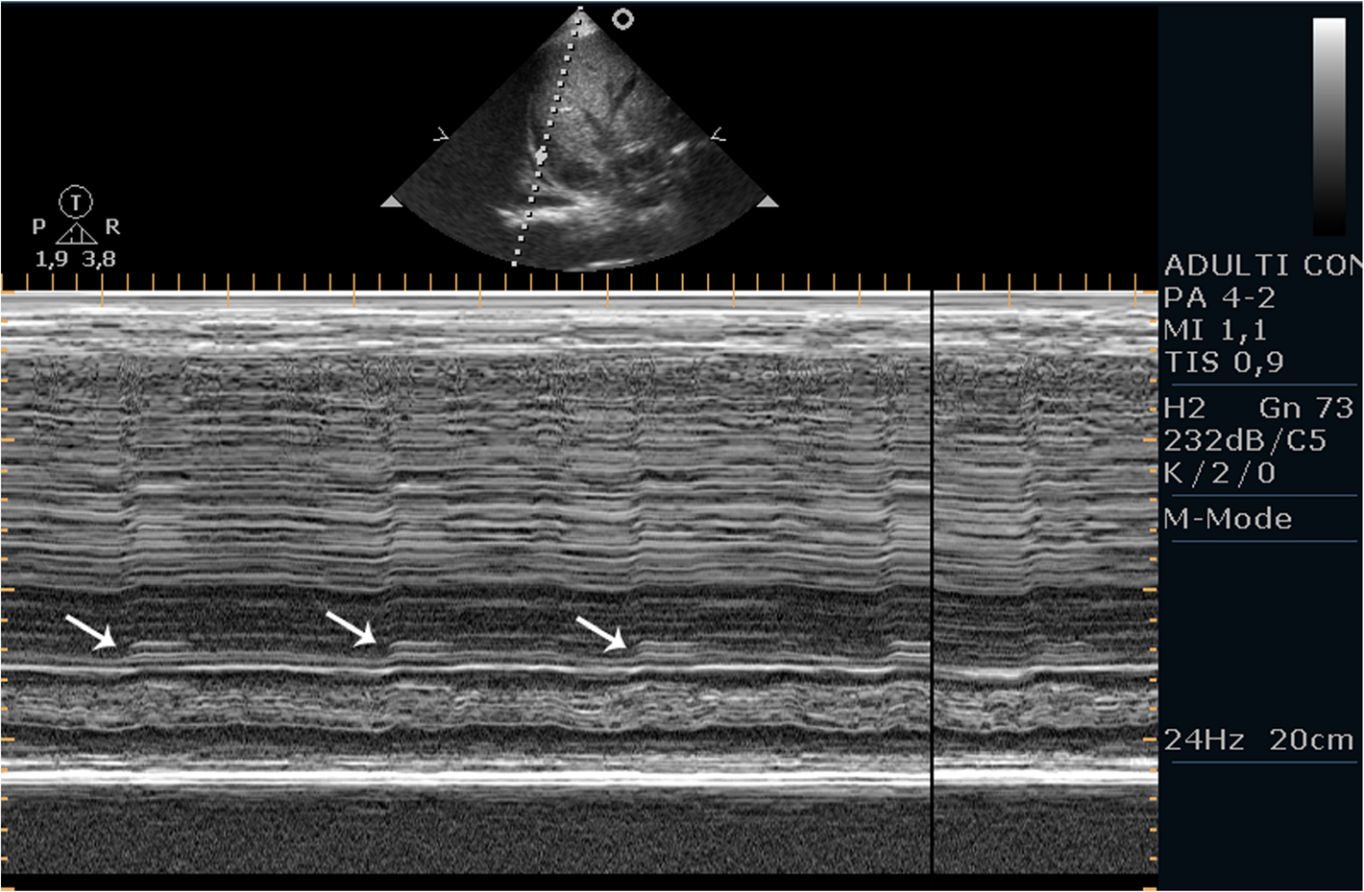
**The pathological right hemidiaphragm of the patient.** There is a dysfunction of the hemidiaphragm with an absent movement (Arrow).

After removal of the laryngeal mask at the end of a general anesthesia, a 50-year-old male patient of 60 kg developed an acute respiratory failure with dyspnea, tachypnea, hypoxia, and hypercapnia. In his medical history, he had been subjected to a liver transplant complicated by wound infection; because of this infection, he has undergone a procedure of VAC therapy of the wound under general anesthesia. Since the cause of this clinical picture was not very clear, the physician in charge decided to perform an ultrasound examination in the operating room. He applied the ‘BLUE protocol’ [4] but without detecting pathological elements capable of explaining the clinical picture; he also evaluated the right and left hemidiaphragms with the patient in the supine position (Philips PA4-2/21422A Sector Array transducer with EnVisor systems, Saronno, VA, Italy). The US approach used to evaluate diaphragmatic kinetics was that proposed by Boussuges et al. [10] in which the liver and the spleen are used as acoustic windows. For the right hemidiaphragm, the probe was placed below the right costal margin in the midclavicular line and directed medially, cranially, and dorsally so that the ultrasound beam could reach perpendicularly the posterior third of the right hemidiaphragm. The left hemidiaphragm was studied from a subcostal probe position between the midaxillary and anterior axillary lines to obtain the imaging of the left hemidiaphragmatic dome. First, the two-dimensional (2D) mode was used to find the best approach and to select the exploration line of each hemidiaphragm; the M-mode was then used to display the movement of the anatomical structure along the selected line. In M-mode, an average of three measurements were performed for each of diaphragmatic excursion (DIA, cm), inspiratory (Tins, s), and expiratory (Tesp, s) time. We observed that the excursions were much larger in the left hemidiaphragm (Figure 1, Additional file 1: VideoClip 1) and that the movement of the right hemidiaphragm could not be measured (Figure 2, Additional file 2: VideoClip 2). On the basis of this information, we then made a diagnosis of diaphragmatic dysfunction, which would probably have been difficult to diagnose without the aid of the bedside ultrasound.

Since the patient was cooperating and capable of protecting his airway, we avoided intubation and moved him to the intensive care, where non-invasive ventilation (NIV) was applied using a ‘full face’ mask (Respironics PerforMax®, Philips, Berlin, Germany). There we began ventilator support with 5 cmH_2_0 of positive end expiratory pressure (PEEP) alone; then, we started to assist the inspiration through a pressure support ventilation (PSV) of 3 cmH_2_O and increased it in increments of 2 cmH_2_O to achieve a 6 to 8 mL/kg expiratory tidal volume (Dragër Evita 4®, Dragër Evita, New York, VA, USA). The peak inspiratory pressure (PSV + PEEP) was set to be less than 25 cmH_2_O. In the following days, the patient’s pulmonary gas exchange gradually improved and was weaned from NIV; after 3 days of ICU stay, the patient was transferred to the ward. Even though he was able to stay in spontaneous breathing without NIV, the right hemidiaphragm continued to be paralyzed (as we said above, recovery from the dysfunction may require a few months and compensatory mechanisms are able to maintain adequate ventilation and gas exchange). No late complications nor other ICU admissions were reported.

## Conclusions

We are prone to believe that in this case, the respiratory failure of our patient was caused by a pre-existent diaphragmatic paralysis, whose physiological compensation had been compromised by the combination of anesthesia and abdominal surgery. To our knowledge, this is the first case report that has shown the usefulness of ultrasonography in detecting diaphragmatic dysfunction as a cause of acute respiratory failure with a subsequent change in patient management. As we said, in the literature, some infections have been associated with phrenic nerve dysfunction (i.e., herpes zoster and Lyme disease) [11,12]. Our patient also had a co-infection with ocular herpes simplex virus; therefore, we cannot exclude that his diaphragmatic failure could actually have been caused by herpes infection. A weak point of the diaphragm ultrasound is the evaluation of the left hemidiaphragm. There are recognized difficulties due to the blocking of sound waves by the presence of gas in the stomach and intestine and the smaller window of the spleen as compared with the liver window [10,13]. In these cases, a lateral approach, although semiquantitative, can be considered. Droffner et al. [14], for example, achieved good visions of the left hemidiaphragm with coronal scans at the midaxillary line just as we did. However, because of these factors, our measurement of the left hemidiaphragm excursion may be underestimated. There are no reference parameters for diaphragm excursion in ventilated patients, so the cutoff for DD in this case report was in agreement with the study of Boussuges et al. [10]: it is the largest study regarding reference values for diaphragmatic motion in which they considered that a measurement lying outside the range of 0.5 to 1.6 should be considered to be abnormal. The left hemidiaphragm excursion measured in this case was 2.78 cm, which is a lower value than that reported by Testa et al. for a normal forced diaphragmatic excursion [15]: they measured a mean resting breath diaphragmatic excursion of 1.8 cm and a forced breath excursion of 7.8 cm. Our value seems to relate more to that of a normal breath than that of a forced compensatory breathing; this may be due to an ineffective compensatory mechanism (underlying cause of the respiratory failure in our patient) or an only semi-quantitative assessment of the diaphragm movements because of the difficulties in US study of the left hemidiaphragm. Ultrasonography can also be used to measure the variations of the thickness of the diaphragm during inspiration, a new and important parameter related to diaphragm function [16-18]. This is a limitation of our case, since we did not detect this measure.

The difficulty in studying DD is partly linked to the cumbersome nature of some diagnostic tools (e.g., fluoroscopy of the diaphragm). US examination provides instead practical functional information on the diaphragm and can also be easily repeated if follow-up is required. Among these, M-mode is the easiest to perform and has a high coefficient of correlation between and within observers [19]. Although the interest in thoracic US is widespread, ultrasound evaluation of diaphragmatic function remains poorly codified. However, extended thoracic ultrasonography may lead to the rapid identification of this feature, still held in little consideration. In conclusion, the use of bedside ultrasonography can be a practical way to evaluate the diaphragmatic function in patients with acute respiratory failure. Ultrasound can affect the diagnosis and the treatment of critically ill patients.

## Consent

Written informed consent was obtained from the patient for publication of this case report and any accompanying images. A copy of the written consent is available for review by the Editor-in-Chief of this journal.

## Additional files

Additional file 1: VideoClip 1. B-mode imaging of the normal left hemidiaphragm of the patient sowing an inspiratory movement. Additional file 2: VideoClip 2. B-mode imaging of the pathological right hemidiaphragm of the patient sowing no inspiratory movements.

## Abbreviations

DD: diaphragm dysfunction
ICU: intensive care unit
NIV: non-invasive ventilation
PEEP: positive end expiratory pressure
PSV: pressure support ventilation.

## References

[1] Solomon DS, Saldana F (2014). Point-of-care ultrasound in medical education-stop listening and look. N Engl J Med.

[2] Mouratev G, Howe D, Hoppmann R, Poston MB, Reid R, Varnadoe J, Smith S, McCallum B, Rao V, DeMarco P (2013). Teaching medical students ultrasound to measure liver size: comparison with experienced clinicians using physical examination alone. Teach Learn Med.

[3] Liebo MJ, Israel RL, Lillie EO, Smith MR, Rubenson DS, Topol EJ (2011). Is pocket mobile echocardiography the next-generation stethoscope? A cross-sectional comparison of rapidly acquired images with standard transthoracic echocardiography. Ann Intern Med.

[4] Lichtenstein D, Mezière G (2008). Relevance of lung ultrasound in the diagnosis of acute respiratory failure: the BLUE protocol. Chest.

[5] McCool FD, Tzelepis GE (2012). Dysfunction of the diaphragm. N Engl J Med.

[6] McAlister VC, Grant DR, Roy A, Brown WF, Hutton LC, Leasa DJ, Ghent CN, Veitch JE, Wall WJ (1993) Right phrenic nerve injury in orthotopic liver transplantation. Transplantation 55:826–30 [PMID: 8475559 doi:10.1097/00007890-199304000-00027] 10.1097/00007890-199304000-00027 8475559

[7] Judson MA, Sahn SA (1996) The pleural space and organ transplantation. Am J Respir Crit Care Med 153:1153–65 [PMID: 8630560 doi:10.1164/ajrccm.153.3.8630560] 10.1164/ajrccm.153.3.8630560 8630560

[8] Afessa B, Gay PC, Plevak DJ, Swensen SJ, Patel HG, Krowka MJ (1993). Pulmonary complications of orthotopic liver transplantation. Mayo Clin Proc.

[9] Feltracco P, Carollo C, Barbieri S, Pettenuzzo T, Ori C (2013). Early respiratory complications after liver transplantation. World J Gastroenterol.

[10] Boussuges A, Gole Y, Blanc P (2009). Diaphragmatic motion studied by M-mode ultrasonography: methods, reproducibility, and normal values. Chest.

[11] Oike M, Naito T, Tsukada M, Kikuchi Y, Sakamoto N, Otsuki Y, Ohshima H, Yokokawa H, Isonuma H, Dambara T (2012). A case of diaphragmatic paralysis complicated by herpes-zoster virus infection. Intern Med.

[12] Derveaujx L, Lacquet LM (1982). Hemidiaphragmatic paresis after cervical herpes zoster. Thorax.

[13] Scott S, Fuld JP, Carter R, McEntegart M, MacFarlane NG (2006). Diaphragm ultrasonography as an alternative to whole-body plethysmography in pulmonary function testing. J Ultrasound Med.

[14] Dorffner R, Eibenberger K, Youssefzadeh S, Puig S, Liskutin J, Papousek A, Grabenwöger F (1998). The value of sonography in the intensive care unit for the diagnosis of diaphragmatic paralysis. Rofo.

[15] Testa A, Soldati G, Giannuzzi R, Berardi S, Portale G, Gentiloni SN (2011). Ultrasound M-mode assessment of diaphragmatic kinetics by anterior transverse scanning in healthy subjects. Ultrasound Med Biol.

[16] Matamis D, Soilemezi E, Tsagourias M, Akoumianaki E, Dimassi S, Boroli F, Richard JC, Brochard L (2013). Sonographic evaluation of the diaphragm in critically ill patients. Technique and clinical applications. Intensive Care Med.

[17] Ferrari G, De Filippi G, Elia F, Panero F, Volpicelli G, Aprà F (2014). Diaphragm ultrasound as a new index of discontinuation from mechanical ventilation. Crit Ultrasound J.

[18] DiNino E, Gartman EJ, Sethi JM, McCool FD (2014). Diaphragm ultrasound as a predictor of successful extubation from mechanical ventilation. Thorax.

[19] Kim SH, Na S, Choi JS, Na SH, Shin S, Koh SO (2010). An evaluation of diaphragmatic movement by M-mode sonography as a predictor of pulmonary dysfunction after upper abdominal surgery. Anesth Analg.

